# Contemporary Review of Multimodality Imaging of the Prostate Gland

**DOI:** 10.3390/diagnostics13111860

**Published:** 2023-05-26

**Authors:** Carli E. Calderone, Eric M. Turner, Omar E. Hayek, David Summerlin, Janelle T. West, Soroush Rais-Bahrami, Samuel J. Galgano

**Affiliations:** 1 Department of Radiology, University of Alabama at Birmingham, Birmingham, AL 35294, USA; 2 Department of Urology, University of Alabama at Birmingham, Birmingham, AL 35294, USA; 3 O’Neal Comprehensive Cancer Center at UAB, University of Alabama at Birmingham, Birmingham, AL 35294, USA

**Keywords:** prostate cancer, prostatic adenocarcinoma, benign prostatic hyperplasia

## Abstract

Tissue changes and the enlargement of the prostate, whether benign or malignant, are among the most common groups of diseases that affect men and can have significant impacts on length and quality of life. The prevalence of benign prostatic hyperplasia (BPH) increases significantly with age and affects nearly all men as they grow older. Other than skin cancers, prostate cancer is the most common cancer among men in the United States. Imaging is an essential component in the diagnosis and management of these conditions. Multiple modalities are available for prostate imaging, including several novel imaging modalities that have changed the landscape of prostate imaging in recent years. This review will cover the data relating to commonly used standard-of-care prostate imaging modalities, advances in newer technologies, and newer standards that impact prostate gland imaging.

## 1. Introduction

The enlargement of the prostate, whether benign or malignant, is among the most common groups of diseases that affect men and can have a significant impact on the length and quality of life. The prevalence of benign prostatic hyperplasia (BPH) increases significantly with age and affects nearly all men as they grow older. Autopsy studies suggest the prevalence to be approximately 8% in the fourth decade of life, 50% in the sixth decade of life, and up to 80% in the ninth decade of life [1]. Malignant hyperplasia is also incredibly prevalent. Other than skin cancer, prostate cancer is the most common cancer among men in the United States, with greater than 250,000 new cases and almost 35,000 deaths attributed to prostate cancer every year [2]. Imaging is an essential component in the differentiation, diagnosis, and management of these conditions. Historically, imaging of the prostate gland has relied on ultrasound (predominantly transrectal but also transabdominal), and the staging of prostate cancer has relied on computed tomography and skeletal scintigraphy. However, multiple modalities have emerged (including several novel imaging modalities) that have changed the landscape of prostate imaging in recent years. Of utmost importance, multiparametric MRI has become the imaging modality of choice for the prostate gland, offering significant improvement in terms of soft tissue resolution when compared to other modalities [3]. This review will cover data behind commonly used standard-of-care prostate imaging modalities, advances in newer technologies, and newer standards that impact the imaging of the prostate.

## 2. Imaging Modalities Used to Evaluate Prostate Pathology

### 2.1. Computed Tomography (CT)

Detailed prostate anatomy is not well-delineated in CT due to poor soft tissue contrast. The central zone may be mildly hyperdense (around 40 to 60 HU), and the peripheral zone may be hypodense (5–10 HU), although it appears largely homogenous in most cases [4]. No specific protocols exist for prostate imaging, and the findings are largely described in the routine venous phase. However, prostate abnormalities generally present as circumscribed mass-like enhancement [5]. 

A CT is not necessary for the diagnosis of BPH, as ultrasound is shown to be equally accurate while eliminating the need for ionizing radiation and providing additional information (discussed in greater detail below). However, prostatic enlargement is a common incidental finding on CT performed for countless other indications and can be defined as a volume greater than 30 cc or as the prostate extends cranially to the level of the pubic symphysis [4]. Further information about the prostate gland morphology, including median lobe growth or mass effect on the bladder, may also aid in the diagnosis of BPH. Additionally, CT offers the potential to evaluate the bladder, where ancillary findings such as distention, trabeculation, or diverticula may support a diagnosis of chronic bladder outlet obstruction. Similarly, the upper urinary tracts may be evaluated for hydroureteronephrosis or other such secondary sequelae and complications. 

**Figure 1:**
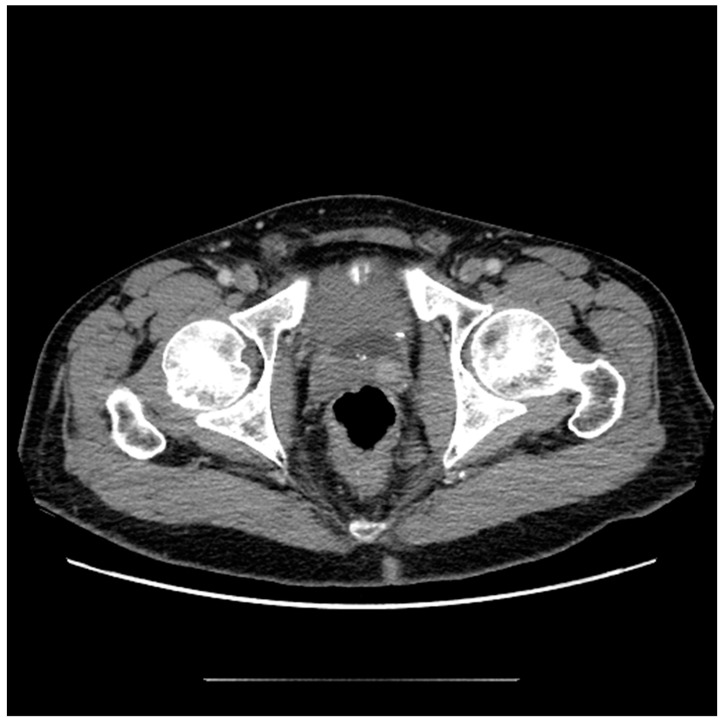
Axial CT image of the pelvis following the administration of intravenous contrast demonstrates focal asymmetric enhancement of the left seminal vesicle, subsequently found to represent prostate cancer at the time of biopsy.

It is widely accepted that CT is insufficient for the diagnosis and monitoring of prostate cancer within the gland due to its inferior soft tissue characterization [6]. The relatively high radiation dose associated with this modality also limits its use as a screening tool. However, although other modalities outperform CT in the diagnostic workup of prostate cancer, it may still have a role. Additionally, with the emerging field of radiomics, additional information can be extracted from CT scans that is not readily apparent in conventional imaging [7,8]. Due to the fact that CT scans are so widely available and frequently performed, familiarity with the appearance and limitations of prostate imaging using CT is useful in order to maximize incidental detection of cancers and inform possible next steps [9]. The ability of CT to identify prostate malignancy depends greatly on the location of the lesion within the gland, as BPH nodules within the transition zone are largely indistinguishable from cancers. For example, in one study, helical CT accurately revealed 58% of the cancer sites in the peripheral zone, whereas nodules in the transition zone were indistinguishable from benign change [10]. Another study showed that although CT was insensitive for the detection of prostate cancer (17.4%), when lesions were considered highly suspicious when using CT, it was 98% specific for malignancy, adding further evidence that suspicious CT lesions warrant further investigation [9]. Additionally, of twenty MRIs with lesions suspicious for clinically significant prostate cancer, fifteen had an accompanying CT correlate of focal intraprostatic hyperenhancement (Figure 1) [5]. 

In recent decades, CT has been less utilized for staging known cases of unfavorable intermediate- or high-risk prostate cancer due to the superior performance offered by other imaging modalities. CT demonstrates relatively poor accuracy in the staging of local disease, capable of identifying seminal vesicle invasion in roughly 69% of cases, with detection rates of extracapsular extension even lower at 24% [6]. 

In evaluating for locoregional and distant metastatic disease, conventional CT underperforms in terms of the identification of nodal metastases, with an accuracy of 72% [6]. This is especially true when CT is compared to newer PET imaging tracers that do not strictly rely on size criteria to identify suspicious nodes [11,12]. Furthermore, while CT performs well in the detection of osseous metastases, the field of view limits its use as a screening tool, and whole-body methods, such as skeletal scintigraphy or PET/CT, are more commonly employed for this diagnostic purpose [13]. 

### 2.2. Ultrasound

The prostate may be imaged transabdominally, transperineally, or transrectally. A transrectal approach (TRUS) is favored in most scenarios due to improved visualization of anatomic structures, including in cases of suspected neoplastic or inflammatory prostate conditions or when an abnormality is encountered transabdominally. Additionally, the transabdominal approach is limited in cases of obesity or inability to maintain bladder distention. The TRUS-guided technique is also the preferred route for conducting biopsies, allowing for biopsy needle placement via both transrectal and transperineal needle puncture approaches. 

For TRUS, an endorectal transducer with a frequency greater than or equal to 8 MHz is inserted into the rectal vault. The transabdominal approach involves placing a transducer above the pubic symphysis, with a distended urinary bladder serving as an acoustic window to the prostate gland. Ultrasound evaluation includes an analysis of the prostatic dimensions, shape, symmetry, echogenicity, and capsular integrity. Doppler examination, when performed, should include a description of overall vascular symmetry as well as the presence of blood flow in the neurovascular bundles outside of the prostate’s capsular boundaries. In the case of suspected carcinoma, TRUS also allows for the evaluation of extracapsular extension and invasion of adjacent structures, including seminal vesicles and neurovascular bundles [14].

The normal prostate gland measures approximately 3 × 3 × 5 cm (average volume 25 cc), where the largest anterior–posterior dimension is the height (H), the transverse dimension is the width (W), and the cephalocaudal dimension is the length (L). Due to its ease of use and high correlation with the actual prostate volume, ellipsoidal volume is the most commonly performed volumetric calculation, which is obtained by multiplying the maximum H × W × L dimensions by 0.524 (or π/6) [15]. This formula is considered valid when dealing with measurements obtained either transrectally or transabdominally, although the European Association of Urology (EAU) suggests measuring the prostatic volume for best accuracy through the use of TRUS only [14]. In addition to prostate volume, the assessment of the relative size of specific zones, including the peripheral and transitional zones, can be determined. If bladder outlet obstruction is suspected due to overall large prostatic volume or intravesical median lobe growth, a transabdominal assessment should be performed to evaluate for post-void residual volumes and bladder morphology. Imaging of the upper urinary tract is also recommended to assess the urinary tract dilation of the ureters and renal collecting systems [16].

Standard TRUS is not routinely used for the diagnosis of prostate cancer due to the fact that prostate cancer tends to often be isoechoic to the benign gland itself. Using color Doppler techniques, increased blood flow reflecting neovascularity may or may not be seen. However, in cases of suspected or known malignancy, ultrasound may be used as an adjunct to assess for extracapsular extension or invasion of local structures, including the neurovascular bundles and seminal vesicles. As a result, this may be particularly useful for treatment planning in patients where prostate MRI is contraindicated or unavailable. As previously stated, this is best performed transrectally for better anatomic visualization. Additionally, new techniques may help aid in the detection of prostate cancer, including elastography and contrast-enhanced ultrasonography [14,17].

One of the most promising advances in the field of ultrasound prostate imaging is the development and validation of high-resolution transrectal microultrasound. This technique operates at 29 MHz, which is significantly higher than standard TRUS and allows for the improved visualization and delineation of the ductal structures within the prostate [18,19]. This approach results in a significantly higher spatial resolution when compared to multiparametric MRI (mpMRI), which has become standard-of-care for prostate imaging but is limited due to the lack of other functional parameters evaluated at the time of MRI. To help guide the findings at the time of microultrasound, a risk assessment system (PRI-MUS) has been developed and utilizes a 5-point scale to convey the risk of cancer analogous to PI-RADS [20]. While MRI–TRUS-fusion-guided biopsies can be utilized to target suspicious lesions identified using MRI, an advantage of the microultrasound approach is that targeted biopsy can be performed at the same time as the diagnostic study, allowing for a more streamlined patient pathway and direct visualization of the suspicious lesion(s) during the biopsy. Additionally, given that this technology can be widely disseminated, performs comparably to MRI in early published reports, and is less expensive than MRI, some have advocated that microultrasound could replace mpMRI in the future as the diagnostic imaging modality of choice for prostatic imaging [21,22].

Transrectal microultrasound has also been combined with several additional ultrasound techniques, including color Doppler ultrasound, Histoscanning, contrast-enhanced TRUS, and ultrasound elastography to form a “multiparametric TRUS” analogous to mpMRI. Given the differences in these technologies, the complementary information gained about enhancement kinetics (similar to MRI dynamic contrast enhancement) and cellular density (similar to MRI diffusion-weighted imaging) draws many parallels to mpMRI. Thus, it is logical that this approach could serve as an alternative to mpMRI, particularly in settings where MRI may not be available. However, a limitation of this approach to date has been the lack of standardization among imaging protocols. Two relatively recent studies compared a multiparametric TRUS approach utilizing grayscale imaging, color Doppler imaging, shear wave elastography, and contrast-enhanced ultrasound to mpMRI and found that the two approaches had comparable detection rates of localized prostate cancer [23,24]. However, additional studies and meta-analyses have not found that this approach outperforms MRI in most cases, and more research is needed with standardized protocols to validate the results [25].

### 2.3. Magnetic Resonance Imaging (MRI)

Multiparametric MRI (mpMRI) of the prostate combines the use of both anatomic and functional pulse sequences [26]. In order to provide standardization in the acquisition, interpretation and reporting of prostate MRI, the European Society of Urogenital Radiology (ESUR) developed the Prostate Imaging Reporting and Data System version 1 (PI-RADS v1) and later version 2 (v2) released in early 2015. Per PI-RADS v2.1, the necessary sequences include T2-weighted imaging, diffusion-weighted imaging (DWI) with an accompanying apparent diffusion coefficient (ADC), and dynamic intravenous contrast-enhanced (DCE) imaging [27]. The most notable differences between v1 and v2 include eliminating the role of MR spectroscopy and minimizing the impact of DCE [28]. 

In a normal prostate, the peripheral zone demonstrates homogenous T2 hyperintensity, while the transition zone is normally more heterogeneous. The surrounding prostatic capsule is homogenously T2 hypointense, in keeping with its fibrous nature. The adjacent seminal vesicles will be uniformly T2 hyperintense, reflecting the fluid they contain. 

While a 3T field strength magnet is considered ideal, a 1.5T magnet may produce acceptable image quality with otherwise optimized imaging protocols for each sequence as part of the mpMRI study. The use of an endorectal versus pelvic coil is institution-dependent, and the decision should factor in the overall image quality achieved [28]. For accurate interpretation of prostate MRI, multiple professional organizations, including the ESUR/ESUI, have urged diligence in both image quality assessment, as well as advanced radiologist training, including additional courses, supervised training, and ongoing performance assessments through comparison with histopathologic outcomes [29]. The PRECISION trial was a major multicenter, randomized study comparing mpMRI/TRUS-fusion-guided biopsies to standard, systematic TRUS-guided biopsies for the detection of prostate cancer, which led to the development of the Prostate Imaging Quality (PI-QUAL) score for evaluation of the quality of mpMRI imaging [30]. The PI-QUAL score is a 5-point scoring system, where a score of 1 indicates that no mpMRI sequences are of diagnostic quality, and a score of 5 indicates that all sequences are of diagnostic quality [31]. This is an essential component of mpMRI in prostate imaging, as poor-quality imaging will yield suboptimal results and an inability to standardize protocols between centers [32,33].

Reflective of their variable pathology, BPH nodules also demonstrate a variable appearance on MRI. For example, depending on the ratio of stromal to glandular components, BPH nodules may be T2 hypointense, as is the case in stromal-predominant nodules reflecting their fibromuscular nature. Conversely, as with glandular-predominant nodules, they are T2 hyperintense, secondary to secretion-filled, ectatic glands [26,34,35]. However, BPH nodules are almost always well-circumscribed on mpMRI, which is in distinction to prostate cancers that arise from the transition zone.

In addition to distinguishing the predominant hyperplastic component, MRI assesses prostatic size and configuration. Estimating prostatic size is important in determining both ideal medical therapies as well as optimal surgical approaches, and MRI has been found to be the most accurate imaging modality when estimating prostate size [36,37]. As previously described, many imaging modalities are sufficient in the estimation of prostatic size; however, MRI demonstrates superiority both in its ability to determine stromal/glandular ratio as well as simultaneously distinguish benign from malignant disease. One limitation of MRI as compared to ultrasound or CT is the limited field of view. While the bladder is well-evaluated through the use of full-field-of-view pelvic sequences, the kidneys, and much of the ureters, are outside of the field of view. Thus, if one desires to evaluate the upper urinary tract, a second imaging modality is often necessary.

Updated guidelines from both the AUA and EAU describe the important role of mpMRI in the evaluation and treatment of prostate cancer [26,38]. In patients with suspected prostate cancer based on an abnormal digital rectal exam or elevated serum PSA, mpMRI is now recommended prior to tissue sampling. This strategy has multiple benefits, including its ability to rule out clinically significant diseases, thereby avoiding unnecessary biopsies and the overtreatment of low-grade cancers [3,39,40]. Additionally, it can identify individuals who would benefit from MRI/US-fusion-guided biopsies in addition to, or in lieu of, systematic biopsies. For example, a recent randomized control trial found that mpMRI in combination with TRUS/MRI fusion biopsies detected 12% more clinically significant cancers and 13% less indolent prostate cancers than systematic biopsy and resulted in 28% fewer biopsies being performed overall [41]. Other work has demonstrated the integral role of mpMRI in active surveillance, with MRI-guided targeted biopsies demonstrating clinically significant prostate cancer in 32% of patients previously classified as eligible for active surveillance on systematic biopsy [42]. Lesions selected for biopsy are categorized via the PI-RADS scoring scheme, which has been validated in several studies and demonstrated moderate interobserver variability [26,43,44]. The most recent PIRADS version (v2.1) was published in 2019 and keeps the overall framework of v2, adjusting some technical parameters and refining a few points of data interpretation [27]. 

**Figure 2:**
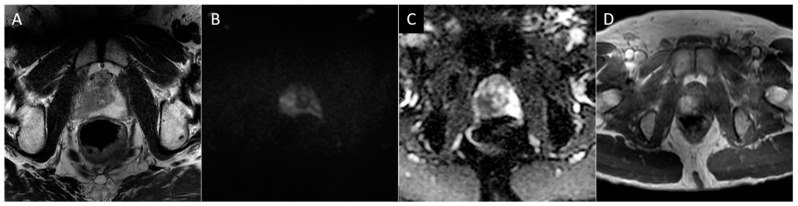
Axial T2 (**A**), diffusion-weighted b2000 (**B**), apparent diffusion coefficient (**C**), and dynamic contrast-enhanced (**D**) images of the pelvis demonstrating a focal ill-defined T2 hypointense lesion in the right midgland posterolateral peripheral zone with corresponding diffusion restriction and asymmetric enhancement, characterized as PI-RADS 5 per PI-RADS v2.1.

To briefly summarize the system, PI-RADS uses a grading scale from 1 to 5, wherein benign-appearing lesions receive a score of 1, and highly suspicious lesions are given a score of 5. The peripheral zone of the prostate is primarily assessed using diffusion-weighted sequences (DWI/ADC), where more convincing diffusion restriction is considered increasingly suspicious. In contrast, the transition zone is primarily assessed using T2-weighted sequences, where hypointense lesions are considered more suspicious as they become less circumscribed, homogenous, and with obscured margins, leading to suspicious lesions often being likened to “smudged charcoal” [28]. In both peripheral and transition zone lesions, maximal lesion diameter determines if a suspicious lesion is categorized as 4 or 5. DCE only impacts the overall scoring of a lesion in situations where a positive DCE can upgrade a peripheral zone lesion from 3 to 4. PI-RARDS v2.1 clarifies that DCE is considered negative if enhancement is diffuse, multifocal, does not correlate to a T2 or DWI abnormality, or if it does correspond to lesions compatible with BPH (Figure 2) [27]. While PI-RADS is also used during the setting of active surveillance, the Prostate Cancer Radiological Estimation of Change in Sequential Evaluation (PRECISE) score has also been developed to help standardize reporting of active surveillance cases and better stratify those who need or can avoid surveillance biopsy [45]. The PRECISE score is based on a 5-point scale where a score of 1 indicates the resolution of suspicious MRI features, 2 indicates a reduction in volume/conspicuity of MRI features, 3 indicates a stable MRI appearance, and scores of 4 and 5 demonstrate a significant increase in volume/conspicuity and/or definite stage progression [45]. Using this standardized approach, this initial study found that PRECISE scores of 1–3 have high NPV and can reduce the need for re-biopsy during active surveillance, while scores of 4–5 have moderate PPV and should be closely monitored or biopsied [45].

The use of PI-RADS is validated only in treatment-naive patients and should not be used for restaging, treatment monitoring, or assessing patients with suspected prostate cancer local recurrence [28]. However, mpMRI is frequently performed in the setting of biochemical recurrence in an effort to detect local recurrence and identify those patients who might benefit from salvage therapy. This is especially important in patients who have previously undergone radiation therapy (RT) due to the relatively high morbidity associated with post-RT salvage therapy techniques [46]. 

Therefore, a panel of experts from international committees, including the European Society of Urogenital Radiology, the European Society of Urologic Imaging, and the PI-RADS Steering Committee, used a combination of the existing literature and expert opinion to develop a standardized approach to mpMRI after whole-gland therapy (RT or radical prostatectomy). Called the Prostate Imaging for Recurrence Reporting (PI-RR) system, the goal of PI-RR was to standardize the acquisition, interpretation, and reporting of post-treatment prostate imaging when evaluating for local recurrence [47]. The scoring of PI-RR is on a 5-point scale, similar to that of PI-RADS, with a higher score correlating to an increased level of suspicion for local prostate cancer recurrence. Overall, mpMRI protocols performed following primary treatment for prostate cancer are similar to pre-treatment image acquisitions. One exception is the recommended inclusion of three orthogonal planes on T2-weighted imaging in post-RP patients, which should include the primary sites of local recurrence, including vesicourethral anastomosis, residual seminal vesicles (if present), and the complete posterior wall of the bladder. Additionally, at least one sequence should be obtained with a large field of view to assess for local nodal or bone marrow abnormalities [47]. The PI-RR scoring in the irradiated gland is based largely upon the diffusion-weighted and post-contrast sequences. T2-weighted sequences are challenging to interpret after RT due to the development of reactive inflammation, glandular atrophy, and fibrosis, resulting in less distinction between benign and malignant lesions [48,49]. However, it is useful in providing anatomic detail and identifying areas that may require closer attention or evaluation through the performance of additional imaging sequences. 

Similar to treatment-naïve prostates, a lesion is suspicious for recurrence if it demonstrates increasingly restricted diffusion (hyperintense on DWI, hypointense on ADC.) Evaluation with DWI is limited in the setting of brachytherapy seed implants due to the susceptibility artifact, in which cases DCE is more heavily relied upon. DCE is considered suspicious in the setting of early enhancement and washout [47]. DCE is subject to false-positive characterization in the early post-treatment period secondary to inflammation and reactive hyperemia; therefore, this sequence should be interpreted with caution less than three months post-RT. In such cases, washout kinetics can help distinguish benign enhancement from malignancy [50]. Similarly, a falsely high ADC signal may be seen in the early post-treatment period and, therefore, a delay in imaging of at least 6 weeks post-RT is recommended [51]. 

After radical prostatectomy (RP), it is recommended to wait at least three months after surgery before imaging. In the post-operative scenario, a PI-RR assessment is most reliant on DCE, with DWI also contributing to the overall score. Although T2-weighted sequences do not contribute to the final assessment, this sequence provides important anatomic detail and helps localize suspicious abnormalities which require further characterization [47]. Frequent sites of recurrence, including the perianastamotic region, vesicorectal space, and, if present, seminal vesicle remnants, should be heavily scrutinized [52]. DCE is highly sensitive for the detection of local recurrence, even when the lesion size is small [53]. Suspicious lesions demonstrate early enhancement and washout kinetics. DWI, while sometimes limited by susceptibility artifact, is accurate in differentiating post-operative inflammation or granulation tissue from recurrent disease, where the former should not demonstrate any restricted diffusion [54]. 

### 2.4. Positron Emission Tomography (PET)

Unlike other modalities, PET imaging in the evaluation of prostate cancer is used exclusively after disease diagnosis, either during initial staging, restaging following biochemical recurrence, or an assessment of treatment response. PET imaging is almost exclusively performed alongside cross-sectional imaging, typically as a PET/CT or, less commonly, PET/MRI. The primary purpose of concomitant cross-sectional imaging is for the anatomic localization of PET tracer activity. Although providing unique and useful information, this modality can be limited due to a larger field of view, lack of IV contrast, and inherent technical limitations. The decision to perform anatomic localization with either CT or MRI may depend on institutional availability as well as the tumor of interest. PET/CT, which is by far more commonly performed, has the advantage of faster image acquisition time, while PET/MRI has improved resolution, which may be beneficial in certain circumstances, including pelvic malignancy. However, it is well known that PET is not as widely available as other imaging modalities, and patients with lower socioeconomic status may not have equitable access to PET and other advanced imaging modalities [55].

In addition to providing physiologic information, PET imaging offers an additional advantage in that its large field of view allows for the characterization of distant metastases in addition to local or regional disease foci. The main disadvantage of PET imaging is the increased dose of ionizing radiation, both due to the radiotracers themselves as well as the larger field of view imaged, often through the use of CT. An additional notable disadvantage of PET imaging is the cost. When considering the use of PET imaging for prostate cancer, the FDA has several approved radiotracers for PET imaging of prostate cancer. As each of these agents has their own strengths and weaknesses, the commonly used agents will be discussed below. 

^18^F-fluorodeoxyglucose (FDG), a glucose analog, is perhaps the most commonly used tracer used in PET imaging, with FDG PET often thought of synonymously with PET imaging. However, FDG demonstrates a decreased sensitivity in the detection of primary prostate cancer [56,57], likely owing to the fact that prostate cancer preferentially uses non-glucose metabolic pathways [56,58]. In addition, there is a significant overlap between benign and malignant prostatic disease with respect to increased FDG activity, thereby limiting its specificity [57]. Given these limitations, FDG has little role in initial staging or restaging in the setting of biochemical recurrence. 

**Figure 3:**
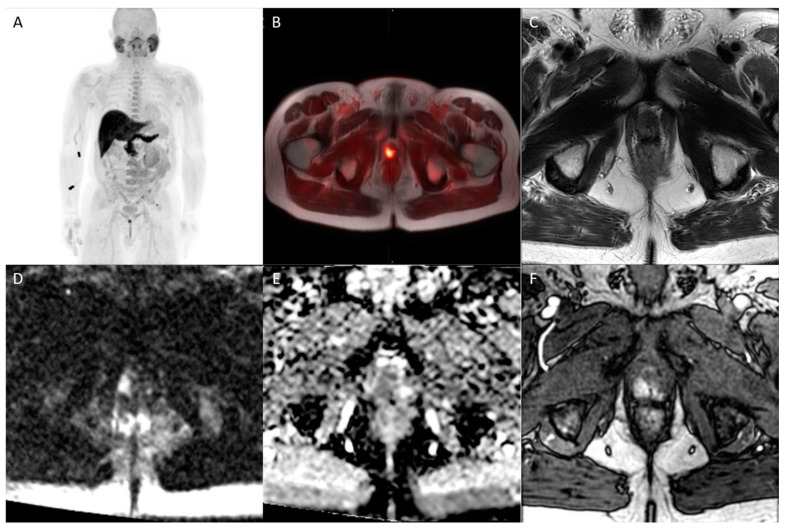
Whole-body maximum intensity projection (**A**), axial fused fluiclovine-PET/MRI (**B**), axial T2 (**C**), diffusion-weighted b2000 (**D**), apparent diffusion coefficient (**E**), and dynamic contrast-enhanced (**F**) images demonstrating focal uptake of radiotracer in the right prostatic apex peripheral zone with corresponding MR defined T2 hypointense lesion with associated restricted diffusion and focal asymmetric enhancement (PI-RADS 5), consistent with prostate cancer.

^18^F-fluciclovine functions as an analog of l-leucine amino acid, which is a transport protein preferentially upregulated in prostate cancer. Unlike many radiotracers used for prostate imaging, fluciclovine has minimal renal excretion, reducing confounding activity in the pelvis from urinary tract activity [59,60]. For initial staging, fluciclovine has limited value in localizing the primary prostate tumor, predominantly due to decreased specificity owing to overexpression in benign etiologies such as BPH and prostatitis [61]. However, fluciclovine performs better in the setting of biochemical recurrence, where updated NCCN guidelines recommend the consideration of fluciclovine PET imaging [62]. In the detection of extraprostatic disease, the tracer performs excellently, with a specificity of up to 100% for disease outside the prostate [63,64]. While the same studies demonstrated diminished specificity for detecting recurrent disease within the prostate itself, this highlights the role of concomitant mpMRI, as previously discussed (Figure 3). 

Both ^18^F- and ^68^Ga-PSMA agents are the newest family of PET imaging radiotracers approved for prostate cancer imaging, with the NCCN adding these agents to their guidelines in 2021. PSMA agents function via binding to the extracellular domain of the PSMA transmembrane glycoprotein that is preferentially overexpressed in prostate cancer cells. Despite the name, PSMA is not entirely specific to the prostate as it is expressed in other cell types such kidney, bowel, and salivary glands. However, there is much less expression within BPH nodules, which offers significant advantages over fluciclovine. 

The recent approval of PSMA makes the precise comparison of these agents to conventional radiotracers difficult. However, for initial staging, evidence suggests that ^68^Ga-PSMA has improved sensitivity for the detection of nodal metastases with excellent specificity in patients with intermediate to high-risk prostate cancer with a detection of 68% of nodal disease and a specificity of 99.1% [65]. To date, PSMA has been demonstrated to have promising data for early disease detection in the setting of biochemical recurrence. In a study of over 200 patients, ^68^Ga-PSMA was able to detect 72.7% of disease in patients with PSA levels between 0.5 and 1 ng/mL and 57.9% of disease with PSA levels less than 0.5 ng/mL [66]. With this improved sensitivity at lower PSA levels, the timeline for earlier detection and treatment of recurrent disease drastically shifts [12]. Additionally, there are early data supporting the use of PET imaging agents, including PSMA PET tracers, for the identification and localization of higher grades of localized prostate cancers [67,68]. Some early work has also been performed analyzing the use of dual-phase PET/CT, with data suggesting that dual-phase imaging at 1 and 2 h post-injection of PSMA radiotracer outperforms single-phase imaging alone [69].

## 3. Treatment Options and Expected Imaging Appearance

### 3.1. Treatment of Benign Disease

The prevalence of BPH has led to the development of numerous treatment options of varying levels of invasiveness. The least invasive interventions consist of medications aimed at symptom management, including alpha-blockers, 5-alpha reductase inhibitors, and even phosphodiesterase inhibitors. As previously discussed, the choice of medication may be at least partially informed by the appearance of the imaging and, specifically, glandular-to-stromal tissue volume ratios. Patients who fail medical management may opt to undergo surgical therapy. The American Urological Association (AUA) guidelines provide a host of validated, evidence-based surgical options, the most common of which will be discussed below. 

**Figure 4:**
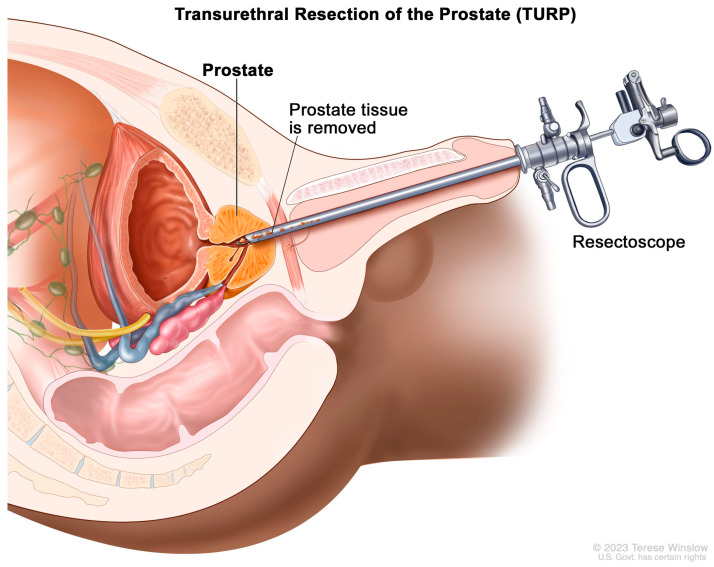
Graphical illustration of the transurethral resection of the prostate procedure. https://nci-media.cancer.gov/pdq/media/images/442342.jpg (accessed on 10 March 2023).

**Figure 5:**
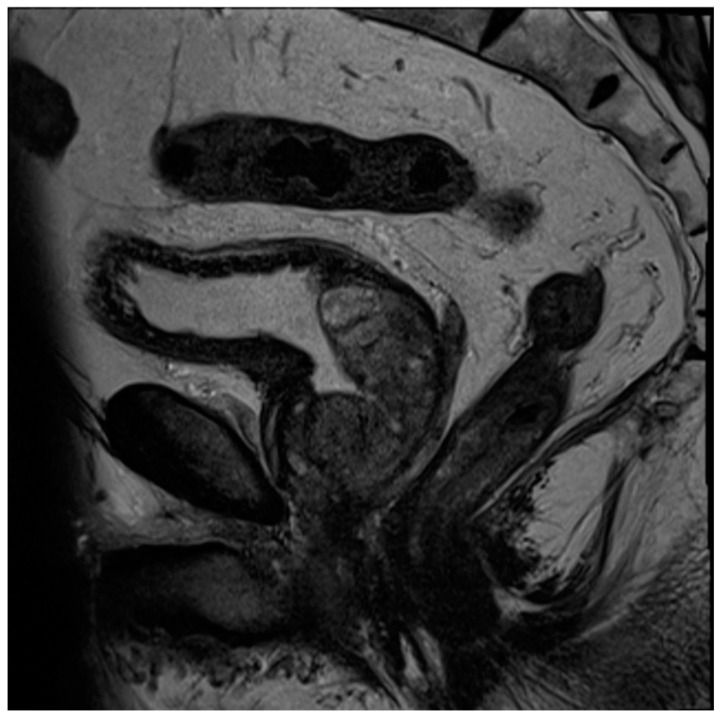
Sagittal T2 image demonstrating post-TURP changes of the prostate. Note the central defect at the level of the prostatic urethra and the relative absence of a median lobe.

The historical mainstay of surgical BPH is the transurethral resection of prostate tissue (TURP) [70]. This involves inserting a thin-loop wire electrode via a cystoscope and resecting adenomatous tissue in the prostatic transition zone that is responsible for bladder outlet obstruction (Figure 4). When using MRI, these changes typically present with an elongated, somewhat irregular appearance of the bladder neck and central prostate gland (Figure 5).

**Figure 6:**
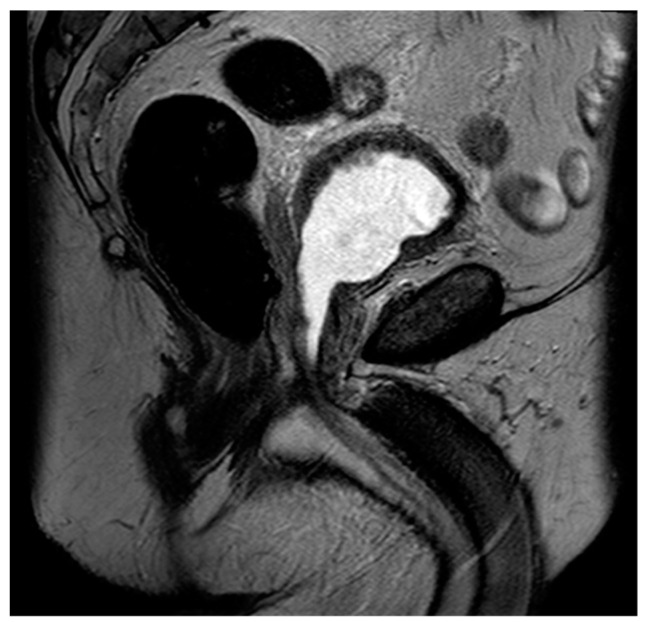
Sagittal T2 image demonstrating post-HoLEP changes to the prostate. As compared to TURP, HoLEP features a wider central defect with less residual peripheral tissue.

Laser enucleation of the prostate (LEP), commonly performed with either a holmium laser (HoLEP) or a thulium laser (ThuLEP), is an ever-increasingly used option for the treatment of BPH. These lasers were first used to vaporize tissue but are now more commonly used to enucleate the entirety of the gland, which is then mechanically morcellated within the bladder in order to facilitate expulsion. One major advantage of LEP, when compared to TURP, is that it is size-independent and can be implemented as a surgical option for glands of varying sizes [71]. When using MRI, LEP procedures look very similar to TURP but often have less residual peripheral tissue and a wider central periurethral defect (Figure 6). There can also be increased T2 intermediate signal and heterogeneity, which can impair the detection of prostate cancers.

**Figure 7:**
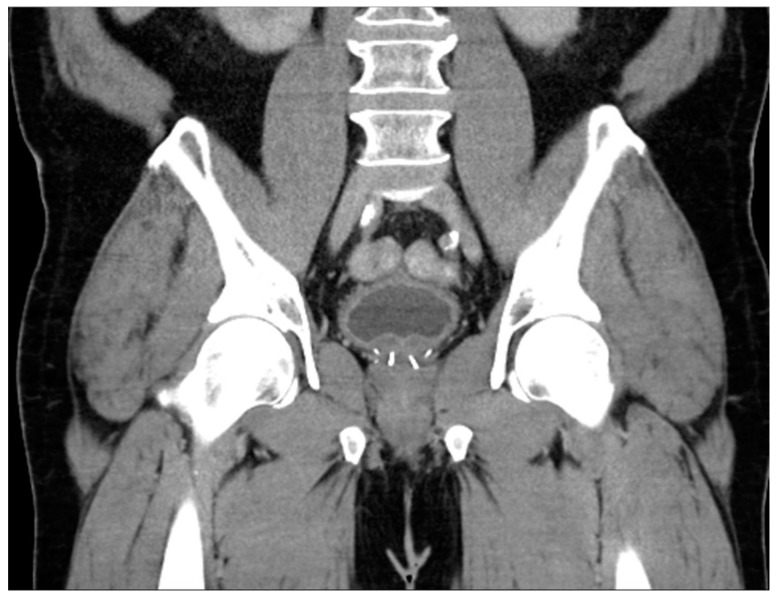
Coronal CT appearance of UroLift device indicated by linear densities within the bilateral prostate gland.

The prostatic urethral lift (PUL), commonly referred to as UroLift, is a clip delivery system that involves the placement of clips under cystoscopic vision that retracts obstructing prostatic tissue. Importantly, the AUA guidelines suggest candidates for this method should not have an obstructive middle lobe, which is easily identifiable in most imaging modalities. The advantages of PUL include the ability to perform in an office without the use of general anesthesia, as well as the preservation of tissue, which provides patients with a higher likelihood of preserving erectile and ejaculatory function [72]. Upon imaging, PUL has an appearance similar to brachytherapy seeds, but there will not be as many present, and they will be confined to the central prostate (Figure 7).

The Rezum system is a relatively new treatment modality for the treatment of BPH that received FDA approval in 2015. It delivers externally heated water as steam into the prostatic parenchyma via a small, thin needle over the course of nine seconds. This ultimately leads to cell death and a reduction in the total tissue volume. Similar to PUL, Rezum’s advantages over more traditional surgical options for BPH include the preservation of sexual function and the potential to be performed in outpatient office settings [73]. 

### 3.2. Treatment of Malignant Disease

Much like BPH, a wide variety of treatment options exist for the treatment and management of men with prostate cancer. The least-invasive approach is known as active surveillance (AS), in which patients with very-low- or low-risk prostate cancer are monitored by a physician with the intent of pursuing definitive treatment if the cancer progresses [74]. Historically, monitoring progression has typically included digital rectal exams and checking PSA every 3–6 months in addition to repeat prostate biopsies every 1–3 years [75]. Recently, mpMRI has been introduced to help guide the frequency and location of biopsies in order to reduce unnecessary biopsies while improving the detection of progression [76,77,78]. Studies have found that when AS is combined with biopsy decisions guided by an annual MRI, there is improved life expectancy, quality of life [79], and overall cost savings when compared to an annual biopsy [80].

**Figure 8:**
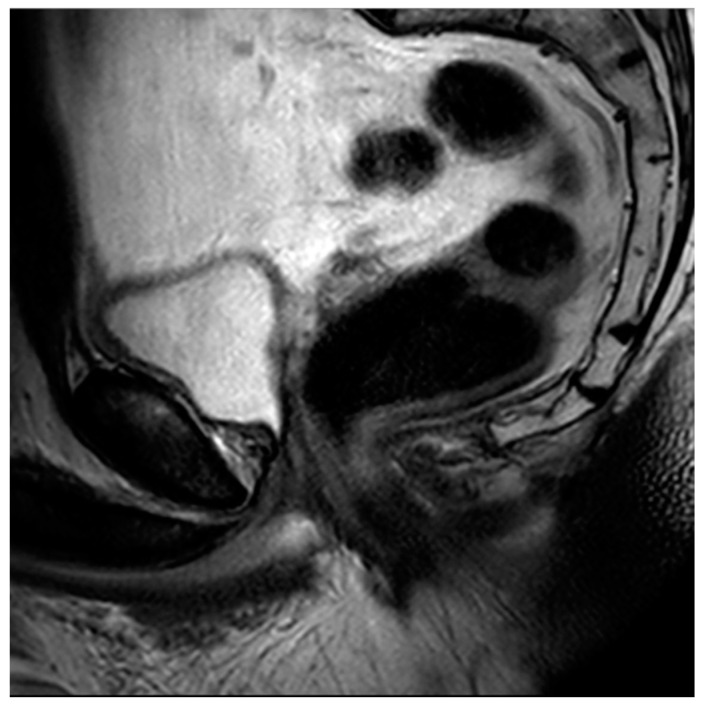
Sagittal T2 image of the pelvis demonstrating a complete absence of the prostate gland with vesicourethral anastomosis following RP.

Radical prostatectomy (RP) is a definitive treatment option for men with prostate cancer in which the prostate and seminal vesicles are surgically removed. The surgical removal of the pelvic lymph nodes may also be offered to men when the chance of nodal metastasis is 2% or higher [81]. Multiple surgical techniques have been described for RP, but the most common approach is robotic-assisted laparoscopic radical prostatectomy (RALP), with up to 90% of all prostatectomies being performed in this manner [82]. Regarding MRI, post-RP changes demonstrate the absence of the prostate gland, with a vesicourethral anastomosis that should have a low T2 signal without early enhancement, nodularity, or restricted diffusion (Figure 8).

**Figure 9:**
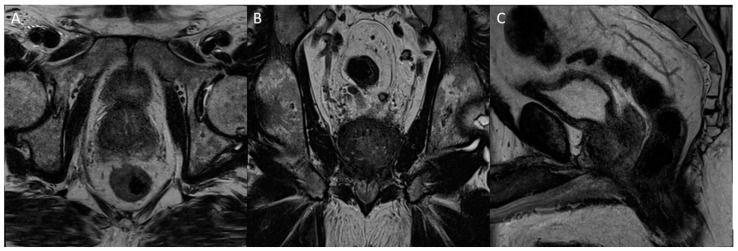
Axial (**A**), coronal (**B**), and sagittal (**C**) T2 images of the irradiated prostate, which demonstrate the loss of features and typical demarcation as well as overall T2 hypointensity, comparable to fibrosis expected post treatment.

Radiation therapy is a treatment modality that can be offered to select patients who seek a non-invasive approach to the treatment of their prostate cancer. The most common form of radiation therapy is external beam radiation therapy (EBRT), in which radiation is delivered to targeted tissues from an outside source through the skin. Brachytherapy is another commonly used method of delivering radiation to cancerous prostate tissue that involves the ultrasound-guided transperineal implantation of tiny radioactive seeds into the prostate. Both temporary or permanent implants, including low dose rate (LDR) and high dose rate (HDR) implants, are another option, which deliver radiation over the course of two months or less than an hour, respectively. The appearance of the prostate following radiation therapy is variable and also potentially confounded by concomitant androgen deprivation therapy being given, but the gland will often appear diffusely T2 hypointense, often combined with the loss of normal features and the demarcation of the peripheral and transition zones (Figure 9).

**Figure 10:**
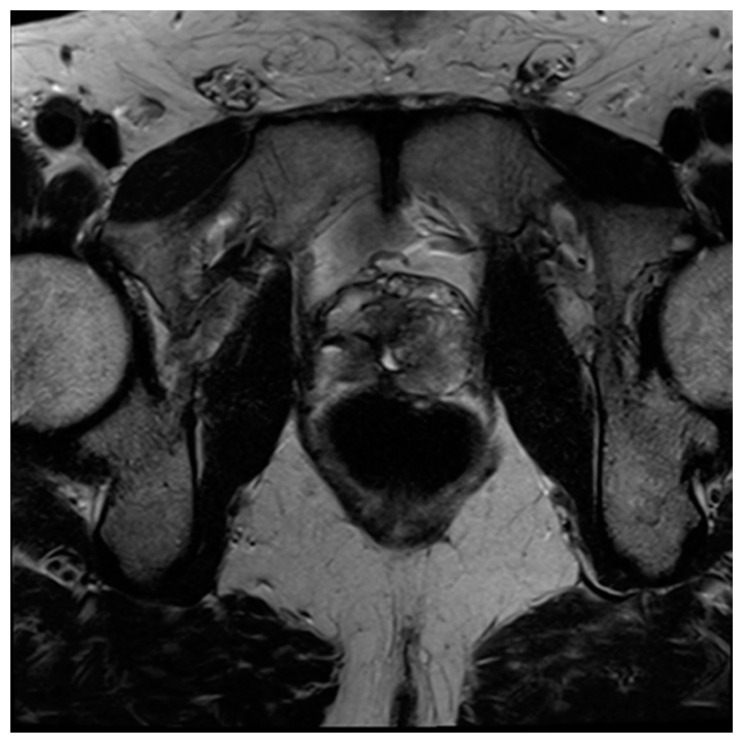
Post-HIFU ablation defect seen on axial T2 MRI as asymmetric atrophy with adjacent fibrosis and architectural distortion, predominantly involving the right hemigland.

Focal ablative therapies are emerging as an alternative treatment option for men with localized prostate cancer, with the aim of sparing benign prostatic tissue in order to reduce the morbidity associated with whole-gland treatment. Of the available focal ablative therapies, high-intensity focused ultrasound (HIFU) has emerged as one of the leading options. This technology involves focusing multiple ultrasound beams on the targeted areas of concern, generating a temperature of at least 55 degrees which ultimately leads to coagulative necrosis [83]. HIFU is particularly well-suited for the treatment of posterior zone tumors but can be challenging with tumors in the anterior gland as the ultrasound energy can scatter as it travels through the prostate tissue or may not effectively reach the target if the focal point falls short of the tissue goal anteriorly. Regarding MRI, post-ablation defects partly depend on whether the whole gland was treated or a hemigland/focal treatment. The treated region demonstrates atrophy, T2 hypointensity, adjacent fibrosis, and the overall architectural distortion of the gland (Figure 10). Newer, more-customizable technologies for focal therapy continue to be developed and validated, including a transurethral ultrasound-guided ablation that utilizes MRI guidance and thermometry throughout the entirety of the procedure and allows for real-time monitoring and contouring of the ablative zone, which is used for both prostate cancer and BPH indications [84]. Additionally, both thermal and electropositive techniques have been reported, which demonstrated success in treating prostate gland pathologies through the use of the transperineal needle approach under real-time imaging guidance, whether MRI- or TRUS-guided.

For the sake of completeness, systemic therapies for patients with advanced and/or metastatic disease are mentioned here in brief. As malignant prostate cancer cells depend on androgens for growth, androgen deprivation therapy (ADT) is a staple treatment in patients with advanced prostate cancer. This can be achieved through chemical castration through the use of drugs that inhibit the enzymes involved in androgen biosynthesis, androgen receptors, or through the suppression of the hypothalamic–pituitary gonadal axis. Castration can also be achieved via bilateral orchiectomy. Finally, a new class of drugs that target prostate-specific membrane antigens (PSMAs) is under development. These are promising agents as PSMAs are highly expressed in prostate cancer cells and only minimally expressed in benign or non-prostatic tissues [85]. Given that many of these treatments are utilized in patients with advanced disease, they can produce a wide spectrum of imaging findings both within the prostate gland and elsewhere in the body.

## 4. Conclusions and Future Perspectives

A wide variety of treatment options exist for men with both benign and malignant prostate disease, and knowledge concerning the expected appearance in imaging studies is essential to radiologists, given the prevalence of prostate pathology. The careful assessment of the clinical scenario, whether pre-treatment or post-treatment, can aid in the detection of benign and malignant findings, the extent of the disease, and post-procedural complications and/or cancer recurrence. mpMRI remains the mainstay of prostate imaging, although other existing and emerging imaging modalities can help assist with problem-solving in certain clinical scenarios. Further research is needed to validate novel imaging modalities of the prostate, as some of these offer significant advantages in terms of cost and feasibility over the use of mpMRI alone.
